# Subtle Patterns of Altered Responsiveness to Delayed Auditory Feedback during Finger Tapping in People Who Stutter

**DOI:** 10.3390/brainsci14050472

**Published:** 2024-05-07

**Authors:** Giorgio Lazzari, Robert van de Vorst, Floris T. van Vugt, Carlotta Lega

**Affiliations:** 1 Department of Brain and Behavioral Sciences, University of Pavia, 27100 Pavia, Italy; giorgio.lazzari01@universitadipavia.it; 2 Centre for Research on Brain, Language and Music (CRBLM), Montreal, QC H3A 1G1, Canada; robertvandevorst@gmail.com (R.v.d.V.); floris.van.vugt@umontreal.ca (F.T.v.V.); 3 School of Communication Sciences and Disorders, McGill University, Montreal, QC H3G 1Y6, Canada; 4 Psychology Department, University of Montreal, Montreal, QC H3T 1J4, Canada; 5 International Laboratory for Brain, Music and Sound Research (BRAMS), Montreal, QC H3T 1J4, Canada

**Keywords:** stuttering, delayed auditory feedback, synchronization-continuation task, tapping

## Abstract

Differences in sensorimotor integration mechanisms have been observed between people who stutter (PWS) and controls who do not. Delayed auditory feedback (DAF) introduces timing discrepancies between perception and action, disrupting sequence production in verbal and non-verbal domains. While DAF consistently enhances speech fluency in PWS, its impact on non-verbal sensorimotor synchronization abilities remains unexplored. A total of 11 PWS and 13 matched controls completed five tasks: (1) unpaced tapping; (2) synchronization-continuation task (SCT) without auditory feedback; (3) SCT with DAF, with instruction either to align the sound in time with the metronome; or (4) to ignore the sound and align their physical tap to the metronome. Additionally, we measured participants’ sensitivity to detecting delayed feedback using a (5) delay discrimination task. Results showed that DAF significantly affected performance in controls as a function of delay duration, despite being irrelevant to the task. Conversely, PWS performance remained stable across delays. When auditory feedback was absent, no differences were found between PWS and controls. Moreover, PWS were less able to detect delays in speech and tapping tasks. These findings show subtle differences in non-verbal sensorimotor performance between PWS and controls, specifically when action–perception loops are disrupted by delays, contributing to models of sensorimotor integration in stuttering.

## 1. Introduction

This paper investigates non-verbal sensorimotor synchronization task performance in people who stutter compared to those who do not by assessing responses to disruptions in action–perception coupling. Childhood-onset fluency disorder, commonly known as stuttering disorder according to the DSM-5 [1], manifests through frequent blocks, repetitions, or prolongations of sounds, syllables, or words, causing interruptions in the natural flow of speech. With a prevalence ranging from 0.3% to 5.6% in the general population, it often co-occurs with other language disorders (for a comprehensive review, see [2]). While the precise origins of stuttering remain elusive [3] and are likely complex, one of the potential underlying causes suggested by contemporary neurobiological research is a generalized deficit in motor timing and the effective integration of sensorimotor processes. Indeed, delayed motor initiation and execution times are frequently observed in people who stutter (PWS)—both in verbal and non-verbal domains [4,5,6,7,8,9,10]. Furthermore, consistent evidence has linked stuttering to differences in the integration of auditory error with ongoing motor articulation [11,12,13]. Supporting this notion, functional neuroimaging studies often reveal aberrant activity patterns within the dorsal auditory–motor network in PWS [14,15,16,17,18,19]. This network connects auditory regions in the posterior temporal lobe with cortical areas such as the parietal, premotor (PMC), supplementary motor (SMA), and primary motor (M1) areas, as well as subcortical regions like the cerebellum and basal ganglia. The dorsal auditory stream is thought to be crucial for integrating auditory and motor information both in speech and in music [20,21,22,23], and it consistently exhibits activity during rhythm perception and production, as well as auditory temporal prediction mechanisms [22,24,25,26,27,28].

The hypothesis that speech disfluencies in stuttering stem from a generalized sensorimotor timing deficit [29,30,31] has been investigated behaviorally by means of finger-tapping tasks. In such tasks, participants are asked to tap their finger in time to a periodic auditory stimulus (a metronome). This line of evidence has revealed conflicting findings when it comes to finger-tapping performance in PWS compared to those who do not stutter (PNS): some investigations reported group differences, indicating decrements in performance among PWS (e.g., [8,29,30,31,32,33,34,35]), while others failed to find discernible distinctions [6,36,37] (see Table 1). These findings, although partly conflicting, collectively suggest there may be impaired coupling mechanisms between external stimuli and neural oscillators [30,38], compromising PWS’ performance in some aspects of perception and production. Such outcomes are significantly associated with motor deficits in initiating and sequencing movements, often accompanied by delays in processing and integrating auditory and motor patterns [32].

One way to study action–perception coupling, which has not yet been used in the literature on tapping in those who stutter, is by introducing delays between movements and the resulting auditory feedback. In the case of speech, for instance, in the delayed auditory feedback (DAF) paradigm, participants’ voice is captured and played back to them after a given delay (e.g., 200 ms). Presumably, in this manner, a discrepancy occurs between the predicted and actual feedback, which triggers compensatory motor responses by the sensorimotor system. Empirically, these adjustments tend to significantly disrupt production, as observed for neurotypical individuals both in speech [43,44,45,46] and music performance [47,48,49,50], increasing with longer delay lengths with a peak at a delay of around 200 ms [45,51,52,53,54]. In PWS, the impact of DAF on speech has been extensively studied. Previous research indicates that while typically fluent speakers delay subsequent speech movements in response to auditory feedback perturbations [55,56,57,58,59], PWS do so significantly less [11,60]. Moreover, they show diminished corrective motor responses to compensate for unexpected auditory timing perturbations in speech [11,60,61]. These findings collectively suggest that adults who stutter have a lower tendency to integrate auditory feedback during real-time monitoring and do not produce appropriate corrective motor responses to auditory perturbation [60,62]. Additionally, such a deficit in multisensory integration [30] aligns with the well-established observation that delayed auditory feedback in speech improves fluency in PWS [63,64,65].

The effects of delayed auditory feedback on speech in stuttering have been extensively documented. However, its impact on non-verbal sensorimotor synchronization abilities in individuals who stutter compared to fluent speakers has, to our knowledge, not been investigated. Indeed, non-verbal sensorimotor tasks, such as tapping tasks, lack the complexity and meaningful auditory feedback inherent in speech [42], potentially influencing the observed group differences in speech DAF studies. As a result, it is currently unknown whether differences between PWS and fluent speakers are restricted to speech or not. Thus, the question remains open as to whether the sensorimotor differences observed in PWS are domain-general or specific to speech. To fill this gap, we focused on DAF during synchronization-continuation tapping tasks (SCT). Nonetheless, investigating the effects of delayed feedback during sensorimotor tapping presents a subsequent challenge. Imagine the participant being asked to tap along with a metronome. When we present a tone (auditory feedback) for each of the participant’s taps, and this tone comes at varying delays after the corresponding tap, the participant could understand the task in two different ways: either to align their physical taps with the metronome (ignoring the auditory feedback timing) or to align their auditory feedback with the metronome (ignoring the physical tap timing). The two objectives cannot be achieved simultaneously if the auditory feedback is delayed with respect to the physical tap. Essentially, these are two different tasks, and to avoid ambiguity, we explicitly instructed participants to do one or the other. In the case where the participant is asked to align their auditory feedback with the metronome (subsequently called “sound instruction”), when the delay increases, they should tap increasingly further ahead of the metronome. In the case where the participant is asked to align their physical tap to the metronome (subsequently called “tap instruction”), when the delay increases, they should not change their tap timing relative to the metronome. To our knowledge, no studies have directly compared these two instructions. Most studies involving delayed feedback during tapping in neurotypical individuals have not explicitly instructed participants to align either the physical tap or the auditory feedback [49,50,51,66,67], in some cases informing participants about the delays [50,67] and in others not [49,66], while in some studies participants were advised to ignore the distracting sound (e.g., [51]). In such studies, a common observation is a reduction in production rate during unpaced conditions, which varies with the length of the delay [49,66,68], along with an increase in timing variability [50,68,69]. However, when Aschersleben and Prinz [70] explicitly instructed participants to synchronize the delayed signal with the pacing one, akin to what we call the sound instruction, participants tapped increasingly further ahead of the metronome with increasing delays (termed negative mean asynchrony, NMA [71]), as expected. This linear increase in asynchrony with increasing delay was evident only in the last trials of each experimental block [70].

The main questions of the current study are: how does delayed auditory feedback (DAF) impact non-verbal sensorimotor synchronization abilities in individuals who stutter compared to fluent speakers? By investigating group differences in non-verbal tapping tasks under DAF conditions, we can infer whether the observed sensorimotor distinctions in PWS extend beyond speech-specific contexts, suggesting a domain-general effect. Additionally, we aimed to investigate whether individuals who stutter process sensorimotor mechanisms differently when aligning physical taps versus auditory feedback with a metronome. This investigation will enable us to address whether the altered sensorimotor integration abilities previously reported in PWS reflect a general impairment in the sensorimotor domain or if they affect specific subcomponents of sensorimotor integration processing. Lastly, we examined whether individuals who stutter and are fluent speakers exhibit different sensitivities to both verbal and non-verbal DAF. To this end, we measured PWS and PNS’ sensitivity to DAF using a delay discrimination task, in which we measured participants’ ability to detect delays of various magnitudes between motor and auditory events (inspired by [72]). Indeed, the ability to discern simultaneity between movement and sound relies on the integrity of both auditory temporal prediction precision and sensorimotor synchronization accuracy. Therefore, we hypothesize a distinct sensitivity to auditory–motor delays between individuals who stutter and those who do not stutter.

## 2. Materials and Methods

### 2.1. Participants

A total of 11 people who stutter (PWS, M = 27.55 years, SD = 3.86, min = 23, max = 35, 6 females) and 13 matched control participants who do not stutter (PNS, M = 26.38 years, SD = 6.10, min = 21, max = 41, 7 females) took part in the study. All participants were right-handed, according to the Edinburgh Handedness Inventory [73]. Subjects reported no language, hearing, and/or neurological deficits (except developmental stuttering). The experimental protocol of all studies was approved by the local ethical committee (Ethical Committee Prot. A10-B58-17B), and participants were treated in accordance with the Declaration of Helsinki. Reading and speech samples collected from participants in the PWS group were used to calculate stuttering severity with the Stuttering Severity Instrument-4 (SSI-4, [74]). Stuttered dysfluencies (sound and syllable repetitions, monosyllabic word repetitions, prolongations, and blocks) in the samples were coded. Accordingly, six participants exhibited very mild stuttering severity, three mild stuttering symptoms, and one severe stuttering. One subject showed no signs of stuttering during the SSI-4 administration but self-identified as a PWS and exhibited stuttering-like dysfluencies during informal conversations outside the laboratory setting. Therefore, the majority of our sample comprises individuals experiencing fairly mild stuttering. Table 2 provides details on the percentage of stuttering events, severity classification, and overall stuttering severity score for each person who stutters (PWS).

### 2.2. Design and Procedure

Participants performed five different tasks. Taken together, the procedure took 1.30 h.

First, they performed a spontaneous (unpaced) tapping task to assess their spontaneous tapping rate (mean and variance) in the absence of a pacing stimulus. Participants were asked to tap as regularly as possible for about one minute at a comfortable, self-chosen pace [30,75].

Second, participants performed a synchronization-continuation tapping task (SCT) without auditory feedback [29,42,76]. In the synchronization phase, participants heard a total of 24 metronome clicks, and they were asked to start synchronizing to the metronome at the fifth click, tapping with their right-hand finger, and then to continue at the same pace after the end of the metronome for an additional eight events. All participants completed 16 trials, each featuring the same metronome inter-onset interval (IOI) of 600 ms (in line with [77]).

**Figure 1:**
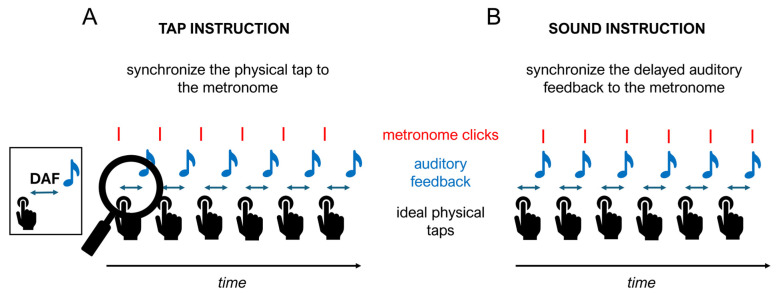
Schematic representation of the two instructions (tap and sound) provided in the synchronization-continuation task (SCT) with delayed auditory feedback (DAF). (**A**) Tap instruction: participants were asked to synchronize their physical finger movements with the metronome, thus disregarding the delayed auditory feedback. (**B**) Sound instruction: participants were asked to synchronize the delayed auditory feedback with the metronome instead of the physical tap. Thus, to perform accurately, participants needed to anticipate their tap based on the delay.

Third, participants undertook a synchronization-continuation tapping task (SCT) with delayed auditory feedback. In each trial, participants performed 32 taps, structured as follows: for the first 8 taps, participants received auditory feedback but no delay (pre-delay clicks). During the subsequent 16 clicks, participants received delayed auditory feedback for each tap, where the amount of delay was constant within a trial and one of the following amounts: 0, 50, 100, 200, or 300 ms. Participants engaged in two variations in the task that differed only in the instructions given, with the order counterbalanced across participants: “tap instruction” (Figure 1A) or “sound instruction” (Figure 1B). In the tap instruction, participants were asked to synchronize their physical finger movements with the metronome, disregarding the delayed auditory feedback. In the sound instruction, they were asked to synchronize the delayed auditory feedback with the metronome. Hence, with the sound instruction, participants needed to anticipate their tap based on the delay to perform accurately, whereas with the tap instruction, they would not need to adjust their tapping based on the delay, which was task-irrelevant. In this manner, the tests were able to assess responsiveness to action–perception delays. Each version involved 4 trials for each delay duration (except for the 0 ms delay, which occurred 8 times), resulting in a total of 24 trials delivered in a randomized order. The rate of the metronome was always set to 600 ms inter-onset interval (IOI).

Then, participants engaged in a tap delay detection task (adapted from [72]) designed to assess their ability to detect delays between motor (tapping) and auditory (tone) events. The objective of this task was to determine which amounts of delay between a tap and tone could be detected by participants. In each trial, participants tapped twice, and for each tap, they heard a tone. The tone was delayed by a variable amount (10, 30, 50, 70, 100, and 200 ms) for one of the taps and not delayed for the other tap (0 ms delay). The tap received the delay randomly in each trial. Participants were asked to verbally indicate which of the two taps they perceived as having a delayed tone (“first” or “second”) across a total of 90 trials.

Finally, participants engaged in a speech delay detection task [13] to offer specific insight into their ability to detect the delay using linguistic materials. Participants produced 150 pairs of the syllable /ba/ separated by a brief pause (“ba… ba”). For one of the two syllables, the participants’ voice was presented back with a delay (15, 25, 40, 60, 90, or 150 ms), and the other was presented with no delay (0 ms). After each trial, participants were asked to verbally indicate which of the two syllables contained the delay (“first” or “second”), and the responses were recorded by the experimenter.

### 2.3. Materials

During the experiment, participants were seated at a table and wore noise-reduction earphones so as not to receive direct sound from tapping or speaking. Taps were recorded using a force-sensitive resistor pad (1.72″ × 1.72″ Interlink FSR) connected to a Teensy microcontroller (version 3.2), and a custom Python script (version 3.5) received the data via the serial port (see [78,79]). This device can simultaneously produce metronome clicks, measure tap time and strength using the FSR sensor, and deliver normal or delayed auditory feedback. The timing information is displayed on the experimenter’s Python interface, where it is saved for offline reading and analysis [79]. The metronome sound was a woodblock sound wave file of 30 ms duration, the same as the tap feedback sound, if present; both are included by default in the Teensy microcontroller [79].

### 2.4. Data Analysis

In the spontaneous (unpaced) tapping task, for each participant, we calculated the interval between subsequent taps (inter-tap interval, ITI) and then took the mean and SD per trial as a measure of preferential tapping tempo. ITIs shorter than 100 ms or exceeding 1.5 times the median of all ITIs in a given trial were excluded from the analysis (following this criterion, 5.93% of ITIs were excluded). We performed a t-test as the main statistical procedure, using the mean-ITI and SD-ITI as dependent variables and group (2 levels: people who stutter, PWS, and people who do not stutter, PNS) as grouping variables. Moreover, due to the shorter time of spontaneous tapping recorded in previous studies [33,39], we conducted the same analyses, filtering ITIs of the first 10 s of the task.

To analyze the SCT without auditory feedback, we computed circular statistical methods [80] using the *circmean* and *circvar* functions in the Python package *SciPy* [81]. For each tap made by the participant, we computed the timing relative to the metronome as the synchronization angle value phi = ((t − m_pre_)/(m_post_ − m_pre_)) × 2π (where t corresponds to each tap made by the participant, m_pre_ to the nearest metronome click preceding each tap, and m_post_ to the nearest metronome click following each tap) [80,82]. We computed this analysis separately for the synchronization and continuation phases. Then, we applied the *circvar* function to the relative phases (angles) within each trial to determine the circular variance, which ranges from 0 (all phases are equal) to 1 (phases are spread uniformly in all directions) in arbitrary units (a.u.) [83,84]. Separately, we computed the circular mean, and we converted the resulting mean angle from phi (in radians) to milliseconds via the formula phi_ms_ = (IOI × phi)/(2π) (where IOI = 600 ms). Thus, we calculated the average of phi_ms within each trial. Next, we performed separate mixed models’ analysis, predicting either the circular mean or the circular variance (as dependent variables) by group (PWS vs. PNS). This was conducted both for the synchronization and continuation phases. All models included random intercepts per subject and per trial to account for both individual differences in response and trials.

In the SCT task with delayed auditory feedback, we computed the same analyses described above, adding the effect of delay as a continuous predictor (from 0 to 300 ms). We also performed two separate analyses for the tap instruction and the sound instruction of the task to account for the different instructions provided to participants across conditions and, thus, the diverse strategies used to complete the task effectively.

All the linear models were fitted using package *lme4* [85] in the R statistical language [86]. Goodness-of-fit was estimated using R^2^ for multilevel models with package *performance* [87,88]. We reported marginal and conditional R^2^ to consider first the variance of fixed effects only and then the total model’s variance (fixed and random effects).

For the tap delay detection task, we computed the accuracy of participant responses (i.e., if they correctly identified the delayed tone), with a value of 1 indicating participants identified the right delayed tone and 0 if they chose the wrong one. Thus, to account for the binary dependent variable, we fitted a logistic regression model, predicting the binary responses using the between-participants factor group (PWS vs. PNS) and within-participants factor delay (considered as a continuous predictor from 10 to 200 ms).

For the speech delay detection task, we were unable to obtain data for 2 PNS and 4 PWS. Consequently, the analyses were performed on the remaining participants (PNS, N = 11; PWS, N = 7) only. Analogously to the tap delay detection task, we performed a logistic regression model with a between-participants factor group (PWS vs. PNS) and within-participants factor delay (continuously coded, 15 to 150 ms) and with a response as a binary dependent variable (1 = participants chose the correct delayed syllable, 0 = participants chose the wrong one). All the logistic regression models were performed with package *stats* in the R statistical language [86].

## 3. Results

**Figure 2:**
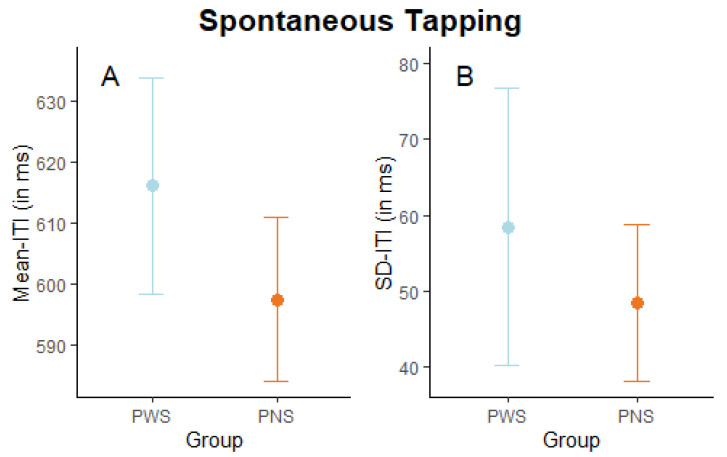
Spontaneous (unpaced) tapping task. Graphical representation of mean-ITI (**A**) and SD-ITI (**B**) in milliseconds by group (people who stutter, PWS, and people who do not, PNS). Dots indicate group averages, and error bars indicate the standard error of the mean and SD ITI.

*Spontaneous (unpaced) tapping*. Analysis of mean-ITI showed no significant effect of group (t (20.65) = −0.37, *p* = 0.716, *d* = 0.15), indicating no significant difference in the preferential tapping tempo between PWS (M = 616.05 ms, SD = 127.64 ms) and PNS (M = 597.47 ms, SD = 117.62 ms), as shown in Figure 2A. Likewise, analysis on SD-ITI revealed no significant effect of group (t (16.01) = −0.48, *p* = 0.639, *d* = 0.20) (PWS (M = 58.46 ms, SD = 60.86 ms) and PNS (M = 48.37 ms, SD = 37.25 ms)), indicating that the two groups are similar also in terms of spontaneous tapping variability (see Figure 2B). In line with previous studies [33,39], in which shorter blocks of spontaneous tapping were recorded, we also computed the mean-ITI and SD-ITI for the initial 10 s of tapping, finding again no significant difference between groups (both *ps* > 0.478).

**Figure 3:**
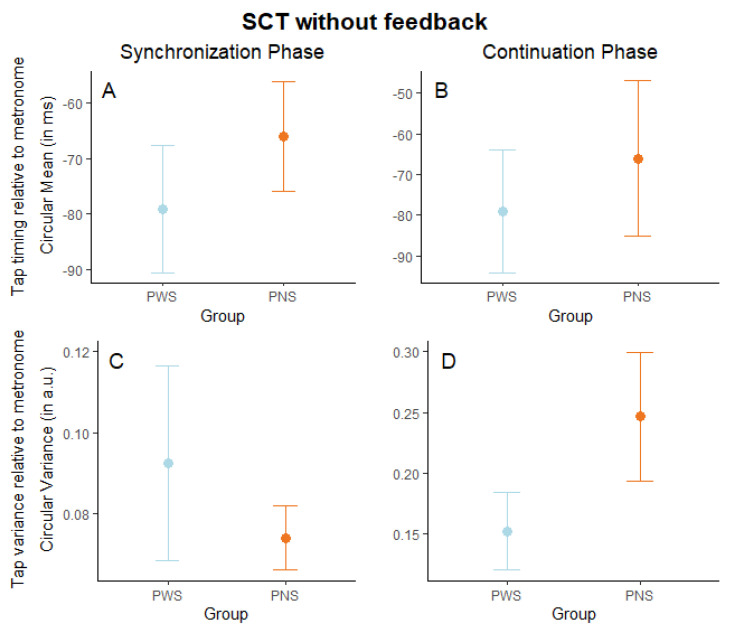
Synchronization-continuation task (SCT) without auditory feedback. Graphical representation of tap timing mean (circular mean in milliseconds) in the synchronization (**A**) and continuation (**B**) phase, and tap time variance (circular variance in arbitrary units) divided into synchronization (**C**) and continuation (**D**) phase, by group (people who stutter, PWS, and people who do not, PNS). Dots indicate group averages, and error bars indicate the standard error of the mean.

*Synchronization-continuation tapping task without auditory feedback*. We first computed a linear mixed model predicting the circular mean by the group (PWS vs. PNS). This analysis revealed no significant group effect in either the synchronization phase (ꭓ^2^ (1) = 0.40, *p* = 0.526; marginal R^2^ = 0.012, conditional R^2^ = 0.713) or the continuation phase (ꭓ^2^ (1) = 1.71, *p* = 0.190; marginal R^2^ = 0.025, conditional R^2^ = 0.330). Although both groups tended to tap ahead of the metronome, as documented in a broad range of prior studies [29,30,32,42,71], these findings indicate that, in a SCT without auditory feedback, PWS’ performance was comparable to that of the PNS both during the synchronization phase (M_PWS_ = −79.09 ms, SD_PWS_ = 49.49 ms; M_PNS_ = −66.02 ms, SD_PNS_ = 63.76 ms) and the continuation phase (M_PWS_ = −47.91 ms, SD_PWS_ = 115.19 ms; M_PNS_ = −6.42 ms, SD_PNS_ = 137.96 ms), as shown in Figure 3A,B. Then, we computed a linear mixed model predicting the circular variance by group. In line with the circular mean, this analysis revealed no significant group effect in either the synchronization phase (ꭓ^2^ (1) = 0.62, *p* = 0.432; marginal R^2^ = 0.011, conditional R^2^ = 0.423) or the continuation phase (ꭓ^2^ (1) = 2.12, *p* = 0.145; marginal R^2^ = 0.042, conditional R^2^ = 0.494) (see Figure 3C,D).

**Figure 4:**
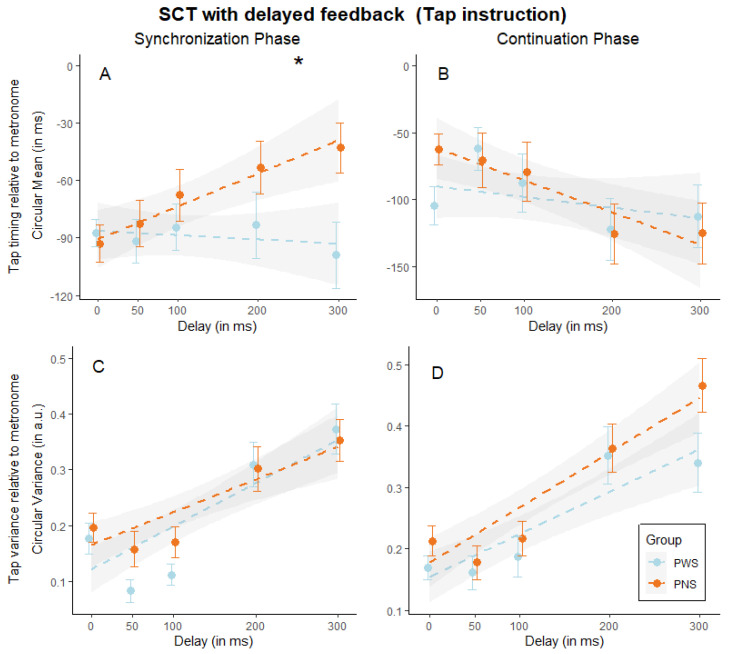
SCT with delayed auditory feedback (tap instruction). Graphical representation of tap timing mean (circular mean in milliseconds) in the synchronization (**A**) and continuation (**B**) phase, and tap time variance (circular variance in arbitrary units) divided into synchronization (**C**) and continuation (**D**) phase, in function of the delay (ranges from 0 to 300 ms) and split by group (people who stutter, PWS, and people who do not, PNS). Dots indicate group averages, and error bars indicate the standard error of the mean, while dotted lines indicate the mixed model fit and the shaded area its standard error. Asterisk indicates a significant interaction group by delay.

*Synchronization-continuation tapping task with delayed auditory feedback*. We first computed a linear mixed model predicting the mean timing relative to the metronome (circular mean) by group (PWS vs. PNS) with tap instruction (when participants were asked to synchronize their physical tap to the metronome). Results are illustrated in Table 3. In both phases, we found a main significant effect of delay, suggesting that even when participants were instructed to synchronize their taps with the metronome while disregarding the auditory feedback, the delayed auditory feedback still influenced their performance (see Figure 4A,B). More importantly, in the synchronization phase, the interaction between delay and group turned out to be statistically significant, providing evidence of group differences in performance at different delays (see Figure 4A). Specifically, in the control group, the delayed auditory feedback significantly influenced the performance, albeit irrelevant to the task, with this influence increasing as the delay increased (ꭓ^2^ (1) = 18.26, *p* < 0.001). On the contrary, in the PWS group, the performance remained stable across delays (ꭓ^2^ (1) = 0.27, *p* = 0.605). When we computed the same models using as the dependent variable the circular variance in the synchronization (see Figure 4C) as well as in the continuation phase (see Figure 4D), we found a main significant effect of delay, indicating that, independently from the group, the different delays affected participants’ performance also in terms of variability.

**Figure 5:**
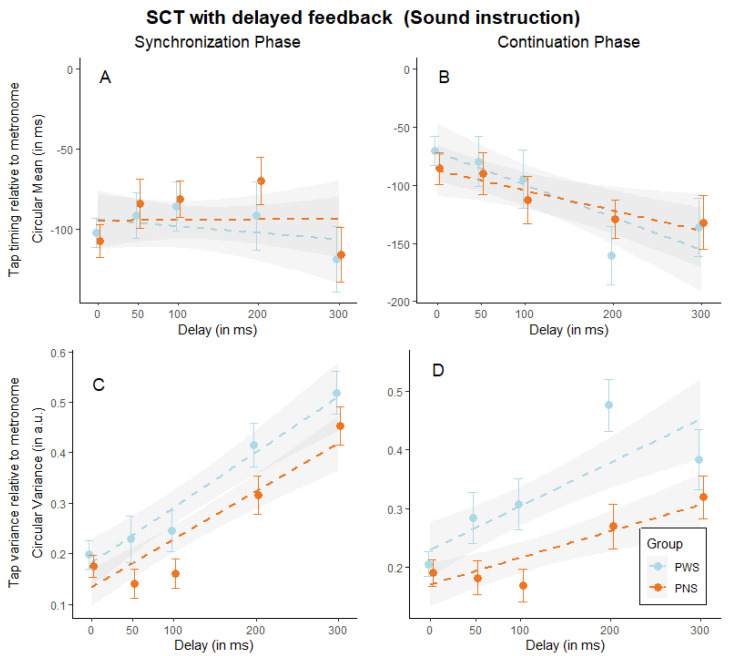
SCT with delayed auditory feedback (sound instruction). Graphical representation of tap timing mean (circular mean in milliseconds) in the synchronization (**A**) and continuation (**B**) phase, and tap time variance (circular variance in arbitrary units) divided into synchronization (**C**) and continuation **(D**) phase, in function of the delay (ranges from 0 to 300 ms) and split by group (people who stutter, PWS, and people who do not, PNS). Dots indicate group averages, and error bars indicate the standard error of the mean, while dotted lines indicate the mixed model fit and the shaded area its standard error.

The same set of analyses was performed in the sound instruction of the task (see Table 4). In the synchronization phase, the results did not show a significant effect of delay. This is an unexpected result, as we predicted participants would exhibit greater anticipation of tapping with increasing delay, as requested by the task instructions (see Figure 5A). We observed this effect in the continuation phase, where the analysis showed a significant main effect of delay, indicating that participants tended to anticipate their tapping more as the delay increased (see Figure 5B). When computing the same models using the circular variance, we found a main significant effect of delay in both phases, indicating that the variability of the participants increases as delay increases (see Figure 5C,D). Moreover, in the continuation phase, the main effect of the group was marginally significant, indicating an overall greater variability for PWS (M = 0.31) compared to PNS (M = 0.22). Finally, the interaction group by delay approached significance, indicating that PWS increased their tapping variability more prominently as the delay increased (ꭓ^2^ (1) = 29.09, *p* < 0.001), compared to the control group (ꭓ^2^ (1) = 18.09, *p* < 0.001).

**Figure 6:**
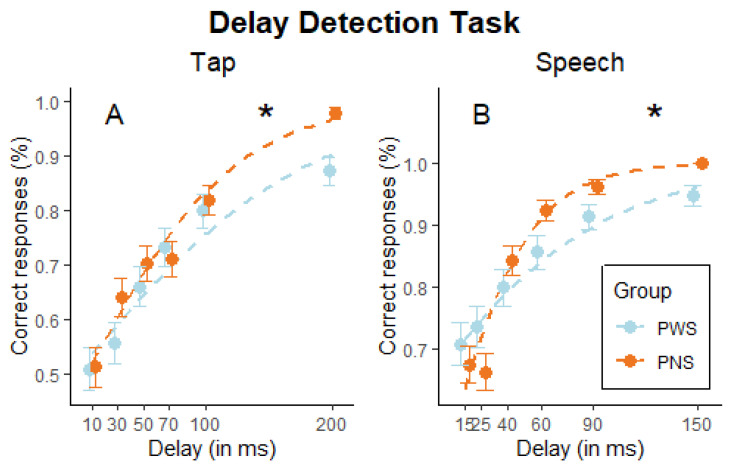
Delay detection task. The percentage of correct responses to delayed sound (**A**) and speech (**B**) is plotted in function of the delay (in milliseconds) and split by group (people who stutter, PWS, and people who do not, PNS). Dots indicate group averages, and error bars indicate the standard error of the mean, while dotted lines indicate the logistic regression fit. Asterisks indicate significant interactions group by delay.

*Tap Delay Detection Task*. The analysis revealed a significant main effect of delay (β = 0.01, z = 7.57, *p* < 0.001), suggesting that overall accuracy increased progressively with the increasing delay. Importantly, this effect was influenced by the group, as evidenced by the significant interaction between delay and group (β = 0.01, z = 2.71, *p* = 0.006), mirroring a more pronounced improvement for PNS (i.e., a steeper logistic regression line) as the delay increased (see Figure 6A). Follow-up analysis showed the group difference held at 200 ms delay (β = 1.94, z = 3.49, *p* < 0.001) but not at any of the other delays (all *ps* > 0.107). No main significant effect of the group (β = −0.13, z = −0.82, *p* = 0.411) emerged.

*Speech Delay Detection Task*. Results of the logistic regression showed a main significant effect of delay (β = 0.02, z = 6.65, *p* < 0.001): the number of correctly identified delayed syllables increases as the delay increases, as expected (see Figure 6B). The main effect of the group was significant (β = −0.71, z = −3.54, *p* < 0.001), indicating that PWS showed a decreased overall number of correct responses. The interaction group × delay (β = 0.02, z = 5.03, *p* < 0.001) was statistically significant: follow-up analysis showed a significant group effect at 60 ms (β = 0.71, z = 2.19, *p* = 0.028) and 90 ms delay (β = 0.92, z = 2.12, *p* = 0.034), compared to all the other conditions of delay (all *ps* > 0.108).

## 4. Discussion

The present study investigated responsiveness to delayed auditory feedback (DAF) during non-verbal sensorimotor synchronization tasks in people who stutter (PWS) compared to controls (people who do not stutter, PNS). Participants performed a synchronization-continuation finger-tapping task with varying amounts of auditory delay. The instruction either required participants to align the feedback with the metronome (sound instruction) or to ignore the feedback and align their physical tap to the metronome (tap instruction). These instructions aimed to explore different aspects of the sensorimotor system: the sound instruction focused on participants’ capacity to use auditory feedback to adjust their motor output adaptively, while the tap instruction examined their ability to disregard auditory feedback when so required and maintain consistent motor performance. Our findings revealed that under tap instruction, neurotypical individuals struggled to fully disregard the auditory feedback, even though it was irrelevant, leading to a measurable impact on their motor performance depending on the degree of delay. In contrast, PWS were less affected by auditory feedback, maintaining consistent motor performance across different delay levels. In the sound instruction, we observed no significant differences between the groups: both PWS and PNS were able to adjust their motor performance in response to delay, which was particularly evident during the continuation phase. Additionally, in a delay detection task, we found PWS were less sensitive to delays, and this held both for speech and for tapping contexts. No group differences were found in spontaneous (unpaced) tapping or during tapping without auditory feedback. These findings suggest that the observed sensorimotor differences associated with stuttering extend beyond the speech domain. These results indicate that PWS diverges from PNS in specific sub-components of sensorimotor integration mechanisms.

A key finding of this study is that, despite being explicitly instructed to disregard auditory feedback while tapping into a metronome, neurotypical individuals’ motor performance is significantly influenced by delayed auditory feedback (DAF). Specifically, the greater the imposed feedback delay, the more participants delay their taps as well, contrary to instructions. Crucially, this pattern was not observed in individuals who stutter (PWS), suggesting they are better equipped to ignore the auditory feedback. This intriguing sensorimotor pattern appears to mirror the discrepancy observed between PWS and PNS in speech-related DAF studies: while PNS tend to delay subsequent speech movements in response to DAF [55,56,57,58,59], PWS demonstrate reduced compensatory motor responses to unexpected perturbations of auditory feedback [11,60,61,89,90]. Furthermore, research suggests that delayed auditory feedback in speech enhances fluency in PWS [63,64,65]. We speculate that this paradoxically beneficial effect on speech observed in PWS may be facilitated by their ability to effectively disregard auditory feedback. Additionally, we may also hypothesize that the diminished sensitivity of PWS to non-verbal DAF, as observed in our delayed detection task, may have specifically aided in their ability to ignore the auditory feedback, thus helping to maintain stable motor performance.

In people who do not stutter (PNS), we did not find expected compensatory behaviors for delayed auditory feedback. When instructed to compensate for delays in the sound instruction, correct performance would necessitated anticipating the taps earlier over time as feedback delays increased. We did not observe this expected effect. Surprisingly, we did observe this pattern during the continuation phase, when the metronome was no longer present, and hence this behavior was no longer required. However, this phenomenon was not evident during the synchronization phase, where their motor performance remained stable across different delays. In previous work on delayed auditory feedback in tapping, Aschersleben and Prinz [70] instructed participants to synchronize the feedback with the metronome while presenting different DAF conditions in separate blocks, intermixed with blocks of no DAF. As expected, their results demonstrated a linear increase in asynchrony with increasing delay; however, this effect was reported only for the final trials of each experimental block, consisting of 180 taps. This suggests that perhaps adapting to delays may require time, and our blocks may have been too short to enable successful adaptation. Indeed, in our experiment, we did not present DAF in blocks. Consistent with this interpretation, we observed the anticipated pattern of results only at the end of each trial, i.e., in the continuation phase. Importantly, we did not observe differences between PWS and PNS. Future research could clarify this issue by having participants synchronize for a longer amount of time per trial instead of the current 24 taps, which might reveal more subtle group differences that elude us here.

Overall, regardless of the specific task instruction—whether participants were directed to attend to the auditory delay or not—the general variability in tapping was significantly influenced by the delay, which increased, as expected, with longer delays in both PWS and PNS. This finding aligns with previous research on neurotypical individuals [50,68,69]. Although not reaching statistical significance, under sound instruction, PWS showed a trend towards greater tapping variability, consistent with a substantial body of evidence [8,29,30,31,32,34,35,41,42].

Another intriguing finding is the general decreased sensitivity to both verbal and non-verbal DAF observed in individuals who stutter (PWS). While diminished sensitivity to verbal DAF has been hypothesized in prior research [91,92], our study presents the first empirical evidence of reduced sensitivity to non-verbal DAF in PWS, both in speech and tapping. This further corroborates our overarching findings, indicating a domain-general sensorimotor impairment in this population. Such tasks as detecting delays tap into the ability to assess simultaneity between movement and sound [72], mirroring a process dependent on the integrity of both perceptual temporal prediction and sensorimotor synchronization accuracy. Deficits in both of these aspects have been previously documented in PWS: altered perception, prediction, and anticipation of rhythmic patterns, as well as ineffective integration of upcoming feedback, are frequently reported [29,93,94]. Despite lacking an explicit rhythmic component (i.e., a beat or metric timing), the tap delay detection task employed in this study required both perceptual and productive sensorimotor processes. Interestingly, Wieland and colleagues [95] highlighted a specific deficit in timing perception in developmental stuttering, suggesting a potential impairment in rhythm perception that could also affect rhythm production. This is consistent with evidence suggesting that the same network of brain areas, including those involved in motor planning and auditory processing, plays a crucial role in both timing production and temporal prediction to guide perception [96,97,98]. In alignment with these findings, evidence points to deficient coupling in PWS of neuronal oscillators affecting communication between the motor and auditory systems [32,34,99], as well as abnormal activity patterns within the auditory–motor network [14,15,16,17,18,19].

No significant group differences were found in spontaneous motor tempo, as measured using an unpaced tapping task. Prior literature on spontaneous motor tapping comparing those who stutter and those who do not appears inconclusive (Table 1). Although certain studies [40] have indicated no notable disparities in voluntary rhythmic tapping movements between PWS and PNS, others have suggested distinctions between the two groups concerning tapping at a comfortable rate [33,39], showing an overall slower spontaneous tapping rate among individuals who stutter. Some crucial methodological differences could have contributed to the observed differences in the current task. For instance, Brown [33] recorded blocks of 15–20 repetitions of spontaneous tapping, and Subramanian and Yairi [39] recorded data for only 10 s. In our task, participants were instructed to tap as regularly as possible for about one minute at a comfortable rate, significantly longer than the durations in the aforementioned studies. To check if the shorter intervals recorded may have led to divergent outcomes, we conducted a follow-up analysis on the first 10 **s** of our spontaneous tapping data, but again, no group differences were found. There are other methodological differences in these prior studies that may contribute to their findings: participants were required to tap faster or slower, but always at a comfortable pace, changing rate every 15–20 repetitions [33], or the motor tapping was changed by combining dominance of hand, number of taps, and simultaneity of taps [39]. These methodological differences may have led to divergent outcomes.

No significant group disparities were detected in the synchronization-continuation task when it was conducted without auditory feedback. This finding aligns with several studies in the literature that found those who do and do not stutter performed similarly in paced tapping [6,36,37]. Similar tapping abilities between PWS and PNS during the presence or absence of an external pacing sound were found by Zelaznik and colleagues [36] and Max and Yudman [37], particularly in the 650 ms IOI condition, in line with our results with a 600 ms IOI. Additionally, Hulstijn and colleagues [6] explored both tapping with one hand and two hands and failed to find differences between groups in pace tapping precision (measured by mean-ITI) and in synchronization phase variance, while concerning the measure of the total variance (i.e., synchronization and continuation phases together), they found differences between groups. Despite statistical differences in computing precision and variance, the results of these studies comprehensively align with ours, thus concluding that rhythmic performance was comparable between PWS and PNS. Taken together, these results do not seem to support the notion that stuttering is linked to overall motor deficits affecting the initiation and sequencing of movements (see also [32]). Instead, our findings suggest that group differences between PWS and PNS in non-verbal timing tasks arise in a specific subset of sensory-motor functions and become evident in tasks that assess how the system deals with delayed feedback, for instance, the synchronization-continuation task with DAF and the delay detection task.

An important limitation of our study is that we were predominantly able to recruit mild stuttering individuals (see Table 2). The results could have been different for individuals who show moderate to severe stuttering. We hope that future work will be able to run similar tests on such samples. Nevertheless, our study was able to detect meaningful differences between individuals who stutter and fluent speakers. Another potential limitation is that, in both tap and speech delay detection tasks, responses were verbally reported by participants and manually recorded by the experimenter. This approach aimed to streamline the procedure for participants, who would otherwise need to press multiple buttons to respond and follow task instructions. Another reason we chose this approach was to maintain compatibility with previous research focusing on sound delay detection, which utilized the same method [72]. However, it is important to acknowledge that this manual recording method may have limitations, such as transcription errors by the experimenter. Future studies could consider automatic recording tools as a feasible alternative.

## 5. Conclusions

In summary, our study sheds light on the nuanced nature of sensorimotor differences in people who stutter. The present study finds that stuttering deficits extend beyond the speech system to more general sensorimotor functioning, but within this more general domain, we find only some aspects are affected (e.g., responsiveness to delay) and others are not (e.g., spontaneous motor tempo). This insight may hold promise for the development of targeted intervention approaches. By identifying and addressing these specific facets of sensorimotor performance that are affected by stuttering, interventions could be tailored to the needs of those who stutter. Moreover, leveraging unaffected areas of the sensorimotor system could enhance the effectiveness of such interventions, potentially leading to more personalized and impactful treatment strategies that capitalize on the characteristics of sensorimotor processing in individuals who stutter.

## Figures and Tables

**Table 1:** Summary of differences or absences in sensorimotor tapping tasks between people who stutter (PWS) and people who do not stutter (PNS).

Phenomenon	Studies Finding Differences in PWS vs. PNS	Studies Finding No Differences in PWS vs. PNS
Slower spontaneous motor performance in PWS vs. PNS	Brown, 1990 [33]; Subramanian and Yairi, 2006 [39]	Webster, 1985 [40]
Higher tapping variability in PWS vs. PNS	Cooper and Allen, 1977 [34]; Falk et al., 2015 [30]; Olander et al., 2010 [41]; Sares et al., 2019 [29]; Slis et al., 2022 [31]; 2023 [32]; Smits-Bandstra et al., 2006 [8]; van de Vorst and Gracco, 2017 [42]; Zelaznik et al., 1997 [35]	Hulstijn et al., 1992 [6]; Max and Yudman, 2003 [37]; Zelaznik et al., 1994 [36]
Greater number of missed taps in PWS vs. PNS	Slis et al., 2022 [31]	Zelaznik et al., 1994 [36]
Greater negative mean asynchrony (NMA) in PWS vs. PNS	Falk et al., 2015 [30]; Sares et al., 2019 [29]; Slis et al., 2023 [32]	Hulstijn et al., 1992 [6]; Max and Yudman, 2003 [37]; van de Vorst and Gracco, 2017 [42]; Zelaznik et al., 1994 [36]
Tempo drift when metronome is discontinued	None	Hulstijn et al., 1992 [6]; Max and Yudman, 2003 [37]; Sares, et al., 2019 [29]; Slis et al., 2023 [32]

**Table 2:** Descriptive statistics of the sample of people who stutter (PWS).

Participant ID	Gender	Age (Years)	SSI Score—Severity	Stuttering Frequency %SS
1	Female	24	17 (very mild)	2.98
2	Female	24	6 (very mild)	0.75
3	Male	27	16 (very mild)	3.79
4	Female	30	35 (severe)	34.29
5	Male	23	14 (very mild)	2.50
6	Female	24	22 (mild)	16.69
7	Female	28	4 (very mild)	0.37
8	Male	32	21 (mild)	7.48
9 *	Female	30	0	0
10	Female	26	20 (mild)	5.78
11	Male	35	10 (very mild)	0.95

* Although the SSI-4 scoring did not indicate stuttering-like events for Participant 9, this participant self-identified as a person who stutters and displays blocks, repetitions, and prolongations during informal conversations. For the sake of clarity, we conducted analyses both including and excluding this participant and found that the results remained consistent regardless of her inclusion or exclusion.

**Table 3:** Results of linear mixed models on circular mean and variance in synchronization and continuation phases, with tap instructions. Each row corresponds to a separate model, with the dependent variable indicated in the leftmost column.

	Circular Mean		Circular Variance	
	Synchronization	Continuation	Synchronization	Continuation
**Delay**	ꭓ^2^ (1) = 7.99, *p* = 0.005	ꭓ^2^ (1) = 10.39, *p* = 0.001	ꭓ^2^ (1) = 68.23, *p* < 0.001	ꭓ^2^ (1) = 107.90, *p* < 0.001
**Group**	ꭓ^2^ (1) = 0.56, *p* = 0.455	ꭓ^2^ (1) = 0.27, *p* = 0.605	ꭓ^2^ (1) = 0.17, *p* = 0.679	ꭓ^2^ (1) = 0.51, *p* = 0.476
**Delay × Group**	ꭓ^2^ (1) = 10.86, *p* < 0.001	ꭓ^2^ (1) = 2.38, *p* = 0.123	ꭓ^2^ (1) = 1.31, *p* = 0.252	ꭓ^2^ (1) = 1.72, *p* = 0.190
**Goodness-of-fit (R^2^)**	Marginal R^2^ = 0.030, Conditional R^2^ = 0.338	Marginal R^2^ = 0.021, Conditional R^2^ = 0.119	Marginal R^2^ = 0.082, Conditional R^2^ = 0.338	Marginal R^2^ = 0.116, Conditional R^2^ = 0.426

**Table 4:** Results of linear mixed models on the circular mean and variance in the synchronization and continuation phases, with sound instructions. Each row corresponds to a separate model, with the dependent variable indicated in the leftmost column.

	Circular Mean		Circular Variance	
	Synchronization	Continuation	Synchronization	Continuation
**Delay**	ꭓ^2^ (1) = 0.28, *p* = 0.595	ꭓ^2^ (1) = 17.67, *p* < 0.001	ꭓ^2^ (1) = 147.32, *p* < 0.001	ꭓ^2^ (1) = 47.13, *p* < 0.001
**Group**	ꭓ^2^ (1) = 0.03, *p* = 0.859	ꭓ^2^ (1) = 0.05, *p* = 0.829	ꭓ^2^ (1) = 1.09, *p* = 0.296	ꭓ^2^ (1) = 2.73, *p* = 0.098
**Delay × Group**	ꭓ^2^ (1) = 0.47, *p* = 0.492	ꭓ^2^ (1) = 0.99, *p* = 0.320	ꭓ^2^ (1) = 0.80, *p* = 0.370	ꭓ^2^ (1) = 2.93, *p* = 0.087
**Goodness-of-fit (R^2^)**	Marginal R^2^ = 0.001, Conditional R^2^ = 0.267	Marginal R^2^ = 0.030, Conditional R^2^ = 0.090	Marginal R^2^ = 0.164, Conditional R^2^ = 0.409	Marginal R^2^ = 0.088, Conditional R^2^ = 0.308

## Data Availability

Data and R code for reproducing our results are available at https://osf.io/gb5j4/?view_only=8b69100d505349eb99ee2f58ff01849a (accessed on 1 May 2024).
